# Some of the Dangers and Disadvantages of Anæsthesia

**Published:** 1884-07

**Authors:** David W. Cheever


### Selections.

### Some of the Dangers and Disadvantages of Anæsthesia.

BY DAVID W. CHEEVER, M. D.

A paper read before the American Surgical Association, April 30, 1884, and published in the Boston Medical and Surgical Journal.

The form of anæsthesia considered in this paper is that produced by sulphuric ether. Unconsciousness of pain in surgical operations is the greatest boon bestowed on man.

What victim of surgery who, under ether, sinks into calm and dreamless sleep, during which his abdomen can be cut open, his bowels taken out, handled, and replaced, his nerves cut, his veins or arteries tied, and his skin sewed up, and who wakes up so absolutely oblivious as to ask, "Are you not ready to begin?" but concedes with gratitude on realizing the result that this is the greatest discovery ever made for the happiness of mankind? Speed of locomotion, electric transmission of speech, the preservation of the printed thought of all the centuries seem at such a moment small in comparason.

Any one about to suffer the agony of an amputation, who was told that if he would take a considerable risk in breathing ether he should feel nothing, would choose the risk and the oblivion.

But the second great quality of this Letheon is that it is almost free from danger. Deaths from ether are rare. Having used it for twenty-six years I have never seen one. The largest statistics give one death in 23,204 cases.

It would take a long list to exhaust the benefits ether has conferred on surgery. The domain of justifiable and beneficent surgery has been greatly enlarged by anæsthesia. The patient seeks an operation with but little dread. He is operated on earlier and better. Many conservative operations are now done which could not be thought of before ether. Many examinations and diognoses can be made with it which could not be made without it. Many simulators and malingerers are detected under its potent spell.

These vast advantages and this wonted safety have masked the disadvantages and obscured the dangers which may arise.

Ether can kill, and there are operations which can be better done without it.

Hitherto all that I have said is trite and axiomatic. It is the object of this communication to point out what is the danger and what the disadvantage in breathing sulphuric ether.

The danger in breathing ether is chiefly from impeded or failing respiration. The heart outlasts the lungs. Suffocation, or coma at the breathing centre, are the causes of death. Temporary paralysis of the palatine muscles causes the stertor or snoring respiration, and the falling back of the *tongue* carries with it the epiglottis, and closes the larnyx. When this condition threatens, the operator opens the mouth, and perhaps draws forward the tongue. When, however, he sees the tip of the tongue out to the incisor teeth he thinks it safe, but in this he is mistaken. The tongue in this position still blocks the entrance of air on account of the falling of the palate, and it is only when the tongue is forcibly drawn out of the mouth that the air passage is opened, and suffocation averted. The larnyx can also be opened by placing from behind the two thumbs under the angle of the lower and pressing forwards. The muscles of mastication being relaxed, the jaw subluxates over the *eminentia articularis* or, rather, on to it, and carries forward the tongue, and with it the epiglotis, and thus opens the larnyx, the mouth being already open.[1]

2. In some persons, and we cannot foresee in whom, the mucous membrane of the throat and air-passages is very sensitive to the irritating fumes of ether. This sensibility first shows itself in a croupy respiration, a spasmodic stridor, followed by an incessant dry cough. Letting in more air relieves the spasm of the glottis, and the narcotism of more profound etherization soon quiets the tickling cough. In many persons there soon follows a new peril from a great secretion of *frothy mucous.* The mouth, pharnyx, and nares bubble with fluid, and soon the same over-secretion pours out in the bronchi, fills the air-passages, and finally approaches a capillary bronchitis. Slow lividity ensues, the arteries spout venous blood, and the patient is on the verge of suffocation, drowned in his own secretions. This is a very tiresome complication. Etherization has to be entirely suspended for a considerable time.

3. Tetanic symptoms are often annoying, and if not relieved by appropriate treatment become dangerous. Obstinate pronation of the fore-arm and wrist, inversion of the thumbs, opisthotonos, boring by boring the head back in the pillow, these are evidences of suffocation, and are relieved by removing the ether, and allowing a few inspirations of air. Once I saw this condition in a child terminate in a convulsion, which, however, was not fatal. A most dangerous complication is *tetanic setting* of the inspiratory muscles of the chest. No air enters; respiration with the diphagram fails to fill the lungs, and the patient dies in true tetanus. I once saw this condition in a middle-aged man. It came on instantly, without warning. The mouth and larnyx were free, tongue out, the position good. Respiration absolutely ceased, but the pulse continued. Artificial respiration, forcibly, by the Sylvester method, restored the breathing, and the chest again inspired. In this case there was no stridor at the glottis.

[1] The relaxation of the muscles in complete anæsthesia being similar to that in death, the manœuvres to open the larnyx were illustrated by the writer on a wet preparation of the head and neck of the cadaver, of which appropriate sections had been made by Dr. Thomas Dwight, professor of anatomy in Harvard University.

The operation was continued and completed without ether, and the patient recovered well. This condition must not be confounded with that where the diaphragm is seen laboriously pumping on a collapsed lung, the glottis being closed by the tongue or by a spasm.

4. *Simple exhaustion* is another source of danger in anæsthesia. Death may occur from slowly failing respiration, so gradual as not to attract notice. Such a case of threatened death from pure debility occurred to me where a feeble woman insisted on an immediate operation for the removal of a cystic tumor of the breast. Sulphuric ether was inhaled quietly, and complete anæsthesia produced, without spasm, frothy secretion, or lividity. The breathing was especially quiet, but both diaghragm and and intercostals were working, the mouth open, and the tongue out. After several imperfect sighing respirations a deathly pallor spread over the face, ears, and neck, the pupils dilated, the jaw fell, the breathing ceased. The pulse continued beating very feebly, the aspect was that of death. Artificial respiration and stimulation restored life. The tumor was removed without ether, and the patient evinced no consciousness until the sutures were being passed. She made a slow but good recovery. A momentary inattention on the part of the surgeon and assistant would have insured a fatal result. A second case like the above occurred to me in operating for a tumor of the jaw. The fauces were free of blood and mucus; there was no obstruction, but breathing gradually ceased. This patient was restored by artificial respiration, and did well. The latter case was being operated on in a chair, and faintness may have contributed to the result, but the former case was lying in an easy position on her side in bed. In neither case was there evidence of pulmonary or cardiac disease.

5. *Disease of the heart* may cause death during the inhalation of ether. Well marked valvular disease, pericarditis and effusion, or a fatty and feeble heart, may contribute to a fatal result, and yet I have repeatedly etherized patients with valvular disease with safety by using caution, and giving them plenty of time. Sulphuric ether is a stimalant to the circulation, and if given slowly, well mixed with air, and not pushed either to lividity or exhaustion, may be given in safety in many cases of heart disease.

A middle-aged man was brought into the hospital with retention and urinary extravasation. Immediate incisions and perineal section were imperative. He was etherized, and as I was about to cut, breathing ceased. The pulse stopped also. Artificial respiration and stimulation revived him. The operation was done without ether. He lived ten days. At the autopsy I found extensive and old valvular disease of the heart.

6. *Apoplexy*, sanguineous or serous, may follow the coma of etherization in the old or in drunkards. Atheromatous vessels may give way under the great congestion produced by full anæsthesia; and the "wet brain" of alcoholism may succeed the stupor of ether.

Having reviewed the dangers, we now pass to the disadvantages of anæsthesia.

Does etherization diminish shock?

Primary shock it does lessen. It removes apprehension, and annuls pain. But it does not remove the secondary shock which follows an operation. Etherization is primarily stimulating, and acts like alcohol in enforcing the heart and filling the pulse.

Secondarily, or long continued, it is exhausting. Long etherization prostrates the patient. A frequent pulse, lowered temperature, drenching sweats, and nausea are the sequences of anæsthesia which lasts more than from half an hour to one hour.

7. One of the marked *disadvantages* of anæsthesia is that it decoys the operator into delay and slowness in his work.

Before ether the skill of the surgeon was estimated by his celerity. A good operator was a brilliant and quick one. The agonies of the patient enforced haste. Every means was used to shorten his sufferings. Now all is changed,—largely to the surgeon's comfort, largely to his increased care and prudence, and as largely to delay, to deliberation over the steps of an operation, and to a too cautious and sometimes puttering execution. The patient may benefit by greater skill, and by more conservatism, but he suffers from prolonged exposure, tedious narcotism, greater secondary shock.

8. Drunkards and steady drinkers succumb slowly to the narcotism of ether. They pass through a long period of excitement and delirium before they get to sleep; they have an equally long and violent delirium on coming out. They are subject during etherization to *persistent* and *recurrent tremors*, which delay an operation or jeopordize its safe execution. It is frequently not until the surgeon has finished that they become quiet and narcotized.

9. *Vomiting* and *lasting nausea* are among the disadvantages of etherization. The larger proportion of patients vomit during anæsthesia or after it, or both. This is especially disastrous in abdominal sections, in operations for hernia, and in section of the cornea and iris.

Vomiting during the operation may expel the vitreous humor.

Vomiting after an operation I have seen extrude a strangulated intestine anew into the hernial sac. Vomiting after an operation is equally liable to disturb the stump of an ovariotomy and bruise peritoneal and omental adhesions, or, in an amputation, to burst the congealed mouths of small vessels, and lead to secondary hemorrhage under the flaps of the stump. Nausea may last two days, and may turn the scale against successful rallying from secondary shock. We know of no agent to arrest it.

10. *Intoxication and delirium* on coming out of ether are especially injurious after a simple, or worse still, a compound fracture has been set. Except in a child, or to settle a diognosis, the setting of broken bones is better done without ether. In nervous women the delirium assumes the form called hysterical. I have seen it last for twenty-four hours.

The following operations are better done without anæsthesia:

Extraction of cataract, already spoken of.

Tracheotomy for disease.

Paracentesis thoracis.

In tracheotomy for croup and diptheria the little sufferer is already partially narcotized with carbonic acid. The cut frightens him no more than the ether would. The incisions through the skin comprise most of the pain. On the other hand, when etherized, he often stops breathing, and the operation has to be done precipitately and not so well. It is absolutely essential, if you open the trachea in a child without anæsthetics, that you have abundant, cool, and competent assistants.

Tapping the chest and pumping off the plural effusion have frequently been perilous, and sometimes fatal, with an anæsthetic. It is a safe operation without it.

There is no reasonable doubt that complete anæsthesia during parturition leads to tardy contraction of the uterus after labor, and hence increases the risk of flooding.

Deaths from inhaling sulphuric ether are rare. It is a noteworthy fact that most of them occur during operations on the rectum, perineum, or in the vagina. The reason is not far to seek. The surgeon is too far removed from the anæsthetic to notice the mouth, the tongue, the respiration, and the diaphragm. An incompetent assistant or a nurse holds the ether sponge, and the patient may die before her peril is discovered. The operator should watch the levator ani muscle, which moves with the diaphragm. This may be called perineal or pelvic respiration.

What are the essentials for giving ether safely?

An empty stomach.

A loose neck.

A free abdomen; no corsets or skirt bands.

Removal of artificial teeth.

An easy, semi-recumbent position.

A sponge wrapped in towels for the ether.

A gag, and forceps for the tongue.

When stertor occurs the patient should be tipped forward, the cheek opened with two fingers, the tongue drawn out, the fauces swabbed. To *insure* safety the surgeon should hear every respiration of the patient.

Anæsthesia from sulphuric ether is of two forms:

1. Primary anæsthesia, which is a moment of confusion coming on after a very few inspirations. At this moment a felon can be opened without pain, and the patient wake at once.

2. Comatose anæsthesia, for prolonged operations. Ether *may* be given almost indefinitely. To relieve the hopeless agony of tetanus I have had it administered for twenty-four hours.

If you would avoid asphyxia, nausea, and headache, and be *safe*, use only the purest anhydrous sulphuric ether.

